# Moderate to severe SARS-CoV-2 infection primes vaccine-induced immunity more effectively than asymptomatic or mild infection

**DOI:** 10.1038/s41541-022-00546-1

**Published:** 2022-10-21

**Authors:** Kayla A. Holder, Danielle P. Ings, Debbie O. A. Harnum, Rodney S. Russell, Michael D. Grant

**Affiliations:** 1 Immunology and Infectious Diseases Program, Division of BioMedical Sciences, Faculty of Medicine, Memorial University of Newfoundland, St. John’s, NL Canada; 2 Eastern Health Regional Health Authority, St. John’s, NL Canada

**Keywords:** Medical research, Adaptive immunity

## Abstract

Hybrid immunity induced by vaccination following recovery from SARS-CoV-2 infection is more robust than immunity induced by either infection or vaccination alone. To investigate how infection severity influenced the strength and character of subsequent vaccine-induced humoral or cellular immune responses against SARS-CoV-2, we assessed humoral and cellular immune responses against SARS-CoV-2 following recovery from infection, vaccine dose 1 and vaccine dose 2 in 35 persons recovered from COVID-19. Persons with polymerase chain reaction or serologically confirmed SARS-CoV-2 infection were recruited into a study of immunity against SARS-CoV-2. Self-reported symptoms categorized them as experiencing asymptomatic, mild, moderate or severe infection based on duration, intensity and need for hospitalization. Whole blood was obtained before vaccination and after first and second doses. Humoral immunity was assessed by ELISA and cellular immunity by ELISpot and intracellular flow cytometry. Responses were compared between groups recovered from either asymptomatic/mild (*n* = 14) or moderate/severe (*n* = 21) infection. Most subjects experienced robust increases in humoral and cellular immunity against SARS-CoV-2 spike (S) protein following 1 vaccination. Quantitative responses to second vaccination were marginal when measured 2.5 months afterwards and moderate or severe infection maintained stronger responses. Polyfunctional CD8^+^ T cell responses were largely restricted to subjects recovered from moderate or severe infection. One vaccine dose triggered stronger immune responses than in a comparable group never infected with SARS-CoV-2, while the second dose produced only minor lasting increases in humoral or cellular responses. Infection history should be considered in planning COVID-19 vaccine administration.

## Introduction

More than 600 million confirmed infections and over six million deaths worldwide have occurred in association with severe acute respiratory syndrome coronavirus-2 (SARS-CoV-2) infection as of August 2022 (https://covid19.who.int). The actual number of infections is undoubtedly much higher than those confirmed, especially with the unprecedented transmission rates of Omicron and its subvariants invoking widespread changes to testing criteria. While most SARS-CoV-2 infections are self-limiting and produce mild coronavirus disease (COVID-19), the course of infection varies from completely asymptomatic, primarily in healthy young individuals, to life-threatening, primarily in older individuals with co-morbidity^1–3^. Where available, vaccination against COVID-19 has reduced rates of transmission and limited the severity of breakthrough infections^4,5^. However, the degree, breadth, and durability of protection provided by vaccines based on ancestral SARS-CoV-2 strains is unclear now and has an even less clear future.

The subunit 1 (S1) domain of SARS-CoV-2 spike (S) protein binds to angiotensin converting enzyme-2 (ACE-2) on host cells to initiate infection and the S2 domain mediates virus fusion with the cell membrane to achieve host cell entry^6^. Neutralizing antibodies (Ab) against SARS-CoV-2 predominantly target the receptor binding domain (RBD) of S1 and prevent binding to ACE-2, thereby physically blocking infection^7–9^. Therefore, the most common approved COVID-19 vaccines are designed to induce immune responses against SARS-CoV-2 S protein. Recent data on the impact third and fourth vaccine doses have on host susceptibility to infection and in vitro Ab-mediated virus neutralization suggest that high Ab levels partially compensate for the increasing variation between vaccine-based and circulating SARS-CoV-2 S antigens^5,10–15^. Breakthrough infections that do occur are generally less severe in recently boosted vaccine recipients, implying that non-sterilizing immunity acting differently than by physically blocking host cell infection can confer protection against illness^16^. Fragment crystallizable (Fc) receptor-dependent non-neutralizing Ab functions and cellular immunity against SARS-CoV-2 are less sensitive to the sequence and structural changes between ancestral and circulating strains of SARS-CoV-2^12,15,17,18^. Thus, there is general consideration that these immune responses are key for protecting against severe illness when the level, affinity, or distribution of neutralizing Ab induced by vaccination or previous infection is insufficient to prevent infection.

Cases of persons completely lacking Ab responses against SARS-CoV-2 who recover from infection with no need for medical intervention empirically illustrate the importance of cellular immunity in controlling SARS-CoV-2 infection^19–23^. Other cases indicating that occult exposure to SARS-CoV-2 induces a cellular immune response that enables viral clearance with no development of symptoms or induction of humoral immunity also imply a key role for cellular immunity^24–26^. Due to priming of their immune systems through selective expansion of SARS-CoV-2 antigen-specific B and T cells, previously infected persons respond more robustly to COVID-19 vaccines than naïve persons^27–33^. Hybrid immunity induced by infection and vaccination is, therefore, stronger than immunity derived from vaccination or infection alone. Whether the level of immune system priming occurring through confirmed SARS-CoV-2 infection varies with the severity of initial infection to an extent that affects subsequent responses to vaccination or infection is a concern for informed SARS-CoV-2 public health policy and vaccine guidance. We compared both infection and vaccine-induced immune responses in two groups, one reporting asymptomatic or mild infection and the other reporting moderate or severe infection. Both groups responded robustly to primary vaccination and marginally to secondary vaccination in terms of response strength and durability. Despite plateauing responses, the group reporting moderate to severe illness maintained stronger humoral and cellular immune responses against SARS-CoV-2 after secondary vaccination. Polyfunctional CD8^+^ T cell cytokine and SARS-CoV-2 S-specific cytotoxic T lymphocyte (CTL) responses were more prominent in persons recovered from moderate or severe infection. Immune response strength may evolve differentially to vaccination or subsequent infection based on initial interactions between SARS-CoV-2 and the immune system.

## Results

### Characteristics of study cohort

To assess the impact of infection severity on infection- and vaccine-induced immunity against SARS-CoV-2, we studied the humoral and cellular immune responses of 35 persons experiencing varied severities of COVID-19 between March 2020 and March 2021. Fourteen persons experienced mild (*n* = 10) or no (*n* = 4) symptoms and 21 experienced moderate (*n* = 17) or severe (*n* = 4) symptoms. Upon enrollment, study participants were asked if they experienced any symptoms prior to being tested, what these symptoms were, how long they lasted and whether they would describe them as mild, moderate or severe. Asymptomatic participants (*n* = 3) were identified through Public Health surveillance and contact tracing after contact with RT-PCR-confirmed cases or through serological testing for anti-S and anti-nucleocapsid (N) protein IgG Ab (*n* = 1). Other individuals were categorized as mild infection if they experienced few symptoms for less than seven days, as moderate infection if they experienced symptoms for more than seven days and as severe infection if they were hospitalized due to COVID-19. The most common symptoms experienced were sore throat, cough, flu-like symptoms (body aches, chills, headache), fever, sinus congestion, and/or runny nose. Whole blood samples were collected from participants between 20 and 394 days post infection (DPI) or days post symptom onset (DPSO), with mean ± standard deviation (SD) of 246 ± 124 days for the asymptomatic/mild group and 214 ± 109 days for the moderate/severe group (Table 1). Positive RT-PCR confirmatory test dates were available for 33/35 participants, with most having contact with a known positive case and experiencing symptoms prior to testing. Antibody testing (anti-S and anti-N IgG) was used to confirm infection and DPSO was used for participants who suspected COVID-19 infection but did not have access to testing offered through Public Health (prior to rapid antigen test availability). The DPSO information for one asymptomatic participant is unknown. There were no significant demographic differences between any groups compared. General demographics of the two groups, vaccines received and mean number of days between vaccine doses are shown in Table 1. Most participants enrolled following the first wave of COVID-19 infections attributed to ancestral Wuhan-Hu-1 SARS-CoV-2 and eight infections were attributed to B.1.1.7.

**Table 1:** Relevant features and categorization of study cohorts.

	Asymptomatic/mild *n* = 14	Moderate/severe *n* = 21	Vaccine controls *n* = 35
Symptoms *n* (%)	Asymptomatic *n* = 4 (11.4) Mild *n* = 10 (28.6)	Moderate *n* = 17 (48.6) Severe *n* = 4 (11.4)	N/A
Mean DPI/DPSI ± SD^a^	245.8 ± 123.5	213.6 ± 108.7	N/A
Median age (IQR) Mean age ± SD	68 (50–72) 58.8 ± 18.1	59 (51–70) 57.4 ± 15.4	62 (47–70) 57 ± 13.7
Female *n* (%)	10 (71.4)	11 (52.4)	21 (60)
Male *n* (%)	4 (28.6)	10 (47.6)	14 (40)
Mean DPV1 ± SD^a^	61.9 ± 14.6	53.1 ± 14.7	61 ± 15.3
Pfizer-BioNTech n (%)	14 (100)	20 (95.2)	33 (94.3)
Moderna *n* (%)	0	1 (4.8)	2 (5.7)
Mean DPV2 ± SD^a^	68.9 ± 29.2	76.9 ± 20.2	69.6 ± 18.2
Pfizer-BioNTech n (%)	12 (85.7)	11 (52.4)	25 (71.4)
Moderna *n* (%)	2 (14.3)	10 (47.6)	10 (28.6)
Mean days between vaccination ± SD	78.1 ± 17.1	67.6 ± 18.7	71.9 ± 13.4

^a^Days post infection or days post symptoms onset at collection (DPI/DPSO); days post first vaccination (DPV1); days post second vaccination (DPV2).

### Natural and hybrid humoral immunity against SARS-CoV-2

**Fig. 1:**
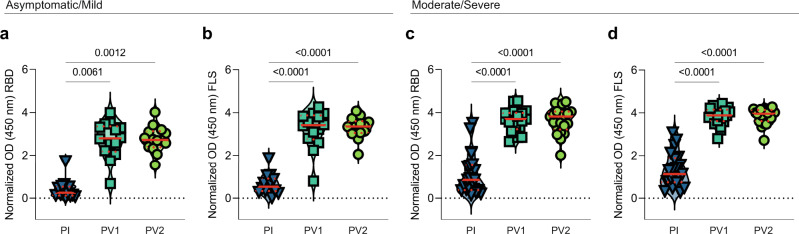
Humoral responses against SARS-CoV-2 RBD and FLS after COVID-19 infection and subsequent vaccination. Plasma was diluted 1:500 and circulating **a** anti-RBD and **b** anti-FLS IgG Ab from those with asymptomatic/mild symptoms (*n* = 14) or **c** anti-RBD and **d** anti-FLS IgG Ab from those with moderate/severe symptoms (*n* = 21) were measured post infection (PI) and after first (PV1) and second (PV2) vaccinations. *P* values were calculated using Friedman’s test with Dunn’s multiple comparisons test and are shown above horizontal lines spanning comparison groups when significant. Red lines bisecting groups represent median (solid) with IQR (dotted).

To compare the strength of humoral responses against SARS-CoV-2 induced in groups with differing severities of infection, circulating IgG Ab against full length S (FLS) protein and its RBD were measured by ELISA post infection, post vaccine 1 and post vaccine 2. Those recovered from asymptomatic or mild infection had low levels of Ab against RBD (Fig. 1a) and FLS (Fig. 1b) that rose significantly after their first vaccination (Fig. 1a, b). Participants who had experienced moderate or severe symptoms had stronger humoral responses against RBD (Fig. 1c) and FLS (Fig. 1d) after infection, which also rose significantly after their first vaccination (Fig. 1c, d). The second vaccination administered ∼2.5 months after the first dose did not significantly increase anti-FLS or anti-RBD Ab levels for either group (Fig. 1). In all cases, mRNA vaccines (Pfizer-BioNTech BNT162b2 and Moderna mRNA-1273) were received for first and second doses (Table 1).

**Fig. 2:**
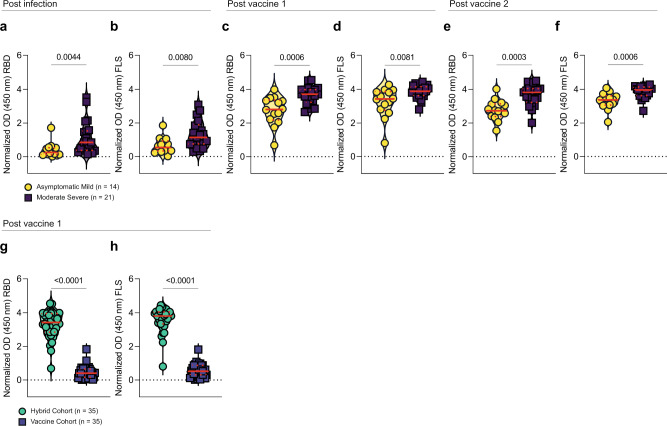
Comparison of humoral responses against SARS-CoV-2 RBD and FLS between asymptomatic/mild, moderate/severe and control groups. Levels of **a** post infection (PI) anti-RBD and **b** PI anti-FLS IgG, **c** PV1 anti-RBD and **d** PV1 anti-FLS IgG and **e** PV2 anti-RBD and **f** PV2 anti-FLS IgG Ab levels were compared between asymptomatic/mild (yellow circle) and moderate/severe (purple square) groups. Levels of **g** anti-RBD and **h** anti-FLS IgG for all previously infected and matched COVID-19-naïve participants PV1 are compared. *P* values, shown above horizontal lines spanning comparison groups, were calculated using the Mann–Whitney *U*-test for (**a**, **c**, **d**, **f**–**h**), and unpaired Student’s *t* test for (**b**, **e**). Red lines bisecting groups represent median or mean (solid) with IQR or SD (dotted) as appropriate.

These data illustrate that IgG responses against SARS-CoV-2 RBD and FLS were significantly greater in the moderate/severe infection group than the asymptomatic/mild infection group post infection (Fig. 2a, b) and post first vaccination (Fig. 2c, d). This difference persisted through the second vaccination (Fig. 2e, f), despite anti-RBD or FLS IgG levels not significantly increasing in either group when measured ∼2.5 months following secondary vaccination (Fig. 1). The combined group of subjects recovered from COVID-19 generated higher anti-RBD and FLS antibody levels following primary vaccination than a group of controls naïve to COVID-19 matched for vaccine type, time between vaccinations and days post vaccination (Table 1) (Fig. 2g, h). Asymptomatic/mild and moderate/severe COVID-19 infection prime the humoral immune system to respond vigorously to primary COVID-19 vaccination, but after ∼2.5 months between doses, secondary vaccination had a marginal impact on circulating anti-SARS-CoV-2 IgG levels in these groups by ∼2 months after vaccination.

**Fig. 3:**
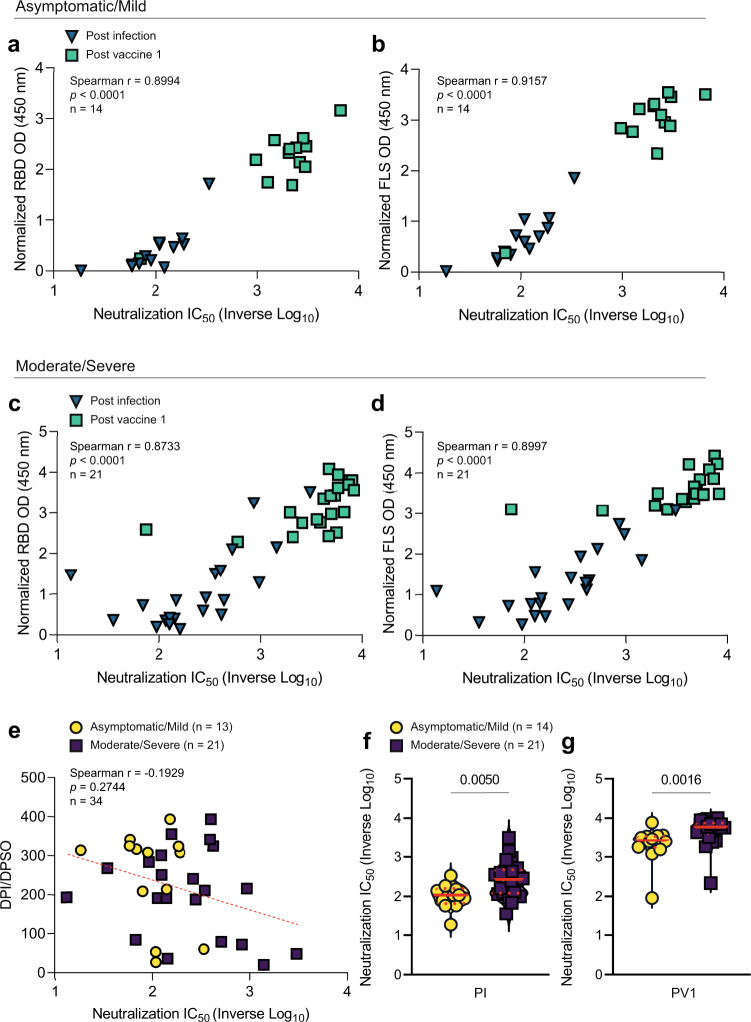
Neutralizing antibody titres and comparisons between groups. Correlations between half-maximal log_10_ inhibitory concentrations (IC_50_) of antibodies required to neutralize rVSV-S infection of Vero cells vs normalized **a** anti-RBD IgG or **b** anti-FLS IgG OD levels after infection (blue chevron) and PV1 (teal square) in the asymptomatic/mild and **c**, **d** moderate/severe groups are depicted. The relationship between days post infection or days post symptom onset at collection (DPI/DPSO) is illustrated in (**e**). Neutralization capacity of Ab activity acquired **f** post infection and **g** PV1 was compared between the asymptomatic/mild (yellow circle) and moderate/severe (purple square) groups. *P* values in **f**, **g** were calculated using the Mann–Whitney *U*-test. Red lines bisecting groups represent median (solid) with IQR (dotted).

We next compared functional activities of anti-S Ab generated by the two groups. Antibodies against RBD and FLS were detectable prior to vaccination in those who had experienced asymptomatic and mild infection and at higher levels in persons experiencing moderate or severe infection (Fig. 2a, b). Neutralization capacity of plasma Ab correlated strongly with anti-RBD and anti-FLS IgG levels measured by ELISA and increased following vaccination in both groups (Fig. 3a–d). Despite the strong correlation overall, one person had low neutralization capacity relative to their absolute IgG Ab levels against RBD and FLS (Fig. 3c, d), illustrating the potential for qualitative differences in the neutralizing activity of Ab against RBD and FLS. There was considerable variability within both groups, which was not simply due to Ab waning as time since infection did not correlate significantly with neutralization capacity (Fig. 3e). Median log_10_ inhibitory titers were significantly higher for the group experiencing moderate/severe COVID-19, both post infection (Fig. 3f, log_10_ 2.00 versus log_10_ 2.44, *p* = 0.005) and after primary vaccination (Fig. 3g, log_10_ 3.33 versus log_10_ 3.67, *p* = 0.002). The higher levels of anti-RBD and anti-FLS antibodies induced in persons experiencing moderate/severe COVID-19 corresponded with greater neutralization capacity both following recovery from infection and after primary vaccination.

**Fig. 4:**
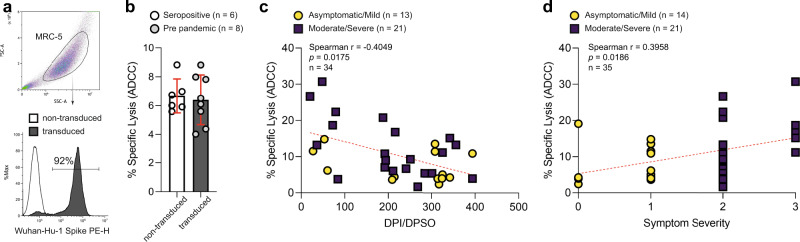
Assessment of antibody-dependent activation of cell-mediated cytotoxicity by plasma collected post infection. **a** MRC-5 cells were gated on forward and side scatter characteristics to illustrate expression of Wuhan-Hu-1 S on non-transduced (white histogram, 0%) and spike-transduced (gray histogram, 92%) MRC-5 cells in a representative histogram. Background NK cell-mediated lysis of **b** non-transduced (white bar) or S-expressing (grey bar) MRC-5 cells mediated either by plasma Ab from SARS-CoV-2-seropositive or seronegative (pre pandemic) individuals, respectively, was measured. Bars represent the mean of individual plasma samples (circles) with standard deviation depicted in red. Natural killer cell-mediated lysis of S-expressing MRC-5 cells enabled by Ab generated after infection was measured by ^51^Cr release (E:T 25:1) and correlation with **c** DPI/DPSO and **d** symptom severity assessed. COVID-19 symptom severity: asymptomatic = 0; mild = 1; moderate = 2; severe = 3. ADCC experiments were performed with three independent PBMC donors and representative plots shown.

Anti-S Ab can physically prevent virus-cell interactions required for infection and can also enact Fc receptor-dependent antiviral functions. To assess the extent to which anti-S Ab produced during infection mediated Ab-dependent cell-mediated cytotoxicity (ADCC), we expressed full length Wuhan-Hu-1 S protein on a human lung fibroblast cell line (MRC-5) (Fig. 4a) to measure the ability of plasma Ab from the two groups to trigger ADCC against these cells. Natural cytotoxicity or non-specific ADCC induced by pre-pandemic plasma Ab against Wuhan-Hu-1 S-expressing MRC-5 cells or by SARS-C0V-2 seropositive plasma Ab against non-transduced MRC-5 cells was <10% (Fig. 4b). Antibodies from 5/13 individuals in the asymptomatic/mild group and 11/21 individuals within the moderate/severe group induced >10% specific killing of Wuhan-Hu-1 S-expressing lung fibroblasts by NK cells from healthy controls (Fig. 4c). Correlation analysis indicates that the ADCC capacity of anti-SARS-CoV-2 S Ab wanes over time (Fig. 4c), while more severe illness is associated with higher ADCC levels (Fig. 4d).

### Circulating natural and hybrid cellular immunity against SARS-CoV-2

**Fig. 5:**
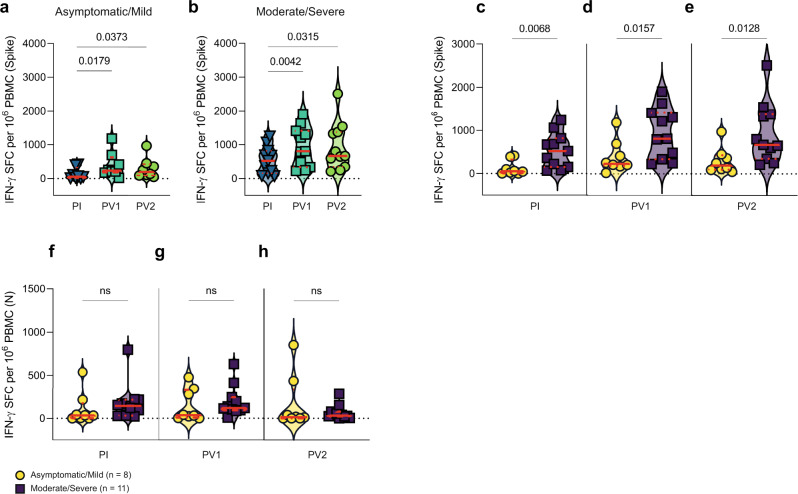
Enumeration and comparison of circulating SARS-CoV-2 S- and N-specific T cells following infection, primary vaccination and secondary vaccination. After 24 h stimulation with S or N peptides, antigen-specific circulating T cells were detected as IFN-γ spot forming cells (SFC). Results from sequential samples obtained after infection, PV1 and PV2 were expressed as SFC/million PBMC and shown for **a** asymptomatic/mild (*n* = 8) and **b** moderate/severe (*n* = 11) groups. *P* values in **a** and **b** were calculated using Friedman’s test with Dunn’s multiple comparisons test and significance is shown above lines spanning the groups compared. Comparisons of IFN-γ SFC/million PBMC after S stimulation between the two groups are shown **c** after infection, **d** PV1, and **e** PV2. Comparisons of N-specific IFN-γ SFC/million PBMC are depicted for samples taken **f** after infection, **g** PV1 and **h** PV2. *P* values in **c**–**h** were calculated using the Mann–Whitney *U*-test. Red lines bisecting groups represent median (solid) with IQR (dotted).

We next compared T-cell responses against SARS-CoV-2 between the groups to investigate their relationship to severity of infection. A subgroup of participants from the groups with asymptomatic/mild (*n* = 8) and moderate/severe (*n* = 11) infection were chosen for these experiments and their general demographics, time between infection and sample acquisition, and time between vaccine doses are shown in Table 2. Circulating T cells responding to SARS-CoV-2 S and N stimulation were measured in PBMC samples collected after infection, after vaccine 1 and after vaccine 2 for each participant. There were few T cells producing IFN-γ in response to SARS-CoV-2 S peptides in the group with asymptomatic and mild COVID-19 (Fig. 5a), which significantly increased after primary vaccination (*p* = 0.0179) but did not further increase after secondary vaccination ~2.5 months later (Fig. 5a). Up to 356 days after infection, participants from the group experiencing moderate and severe infection had circulating T cells producing IFN-γ in response to S peptides (Fig. 5b). As with the asymptomatic/mild group, circulating S-specific T cells increased in frequency after primary vaccination (*p* = 0.0042) but did not further increase after secondary vaccination (Fig. 5b). Circulating T-cell responses against SARS-CoV-2 followed the same trend as humoral responses in groups experiencing asymptomatic/mild or moderate/severe COVID-19 in that secondary vaccination had a marginal effect on the frequency of circulating T cells against S peptides.

**Table 2:** Relevant features of functionally studied cohorts.

	Asymptomatic/mild *n* = 8	Moderate/severe *n* = 11
Symptoms n (%)	Asymptomatic *n* = 2 (10.5) Mild *n* = 6 (31.6)	Moderate *n* = 8 (42.1) Severe *n* = 3 (15.8)
Mean DPI/DPSI ± SD^a^	238.8 ± 118.7	189.8 ± 119.2
Median age (IQR) Mean age ± SD	68 (53.7-70.5) 59.2 ± 18.4	59 (52-66) 58.1 ± 12.5
Female *n* (%)	5 (62.5)	6 (54.5)
Male *n* (%)	3 (37.5)	5 (45.5)
Mean DPV1 ± SD^1^	56.6 ± 13.2	50.9 ± 14.6
Pfizer-BioNTech n (%)	8 (100)	10 (90.9)
Moderna *n* (%)	0	1 (9.1)
Mean DPV2 ± SD^1^	71.8 ± 31.1	78.9 ± 21.3
Pfizer-BioNTech n (%)	7 (87.5)	5 (45.5)
Moderna *n* (%)	1 (12.5)	6 (54.5)
Mean days between vaccination ± SD	76 ± 16.5	64.7 ± 23.2

^a^Days post infection or days post symptoms onset at collection (DPI/DPSO); days post first vaccination (DPV1); days post second vaccination (DPV2).

Although T cell IFN-γ production in response to S peptides after vaccination was consistent in both the asymptomatic/mild and moderate/severe cohorts, the magnitude of the response was significantly different between groups. The group experiencing moderate or severe COVID-19 symptoms had a higher frequency of T cells responding to S peptides than those with asymptomatic or mild infection pre-vaccination (Fig. 5c, *p* = 0.0068), after primary vaccination (Fig. 5d, *p* = 0.0157) and after secondary vaccination (Fig. 5e, *p* = 0.0128). In contrast, no significant difference in the frequency of circulating T cells producing IFN-γ in response to SARS-CoV-2 N peptides was detected between the groups experiencing asymptomatic/mild versus moderate/severe infection either before or after vaccination (Fig. 5f–h). As with humoral anti-S IgG responses, moderate/severe infection induced a significantly stronger cellular immune response than asymptomatic/mild infection that persisted through two rounds of vaccination, despite not increasing in either group after secondary vaccination.

### Inducible natural and hybrid cellular immunity against SARS-CoV-2

**Fig. 6:**
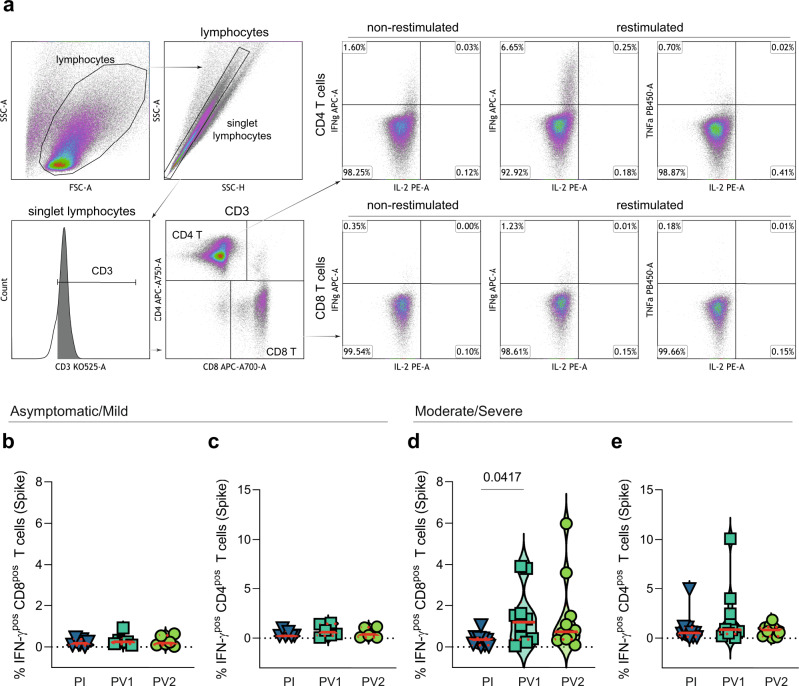
Effector CD4+ and CD8+ T-cell responses to SARS-CoV-2 S peptides following 7 day in vitro stimulation. **a** Lymphocytes were identified by scatter characteristics and doublet exclusion (singlet lymphocytes) then T cells identified as CD3^+^ lymphocytes and subsets distinguished by either CD4 or CD8 expression. Bivariate analysis identified subsets of CD4^+^ T cells (top panel) and CD8^+^ T cells (bottom panel) producing TNF-α, IFN-γ and IL-2 after restimulation at day 7 and background signals from non-restimulated cells were subtracted. Percentages of IFN-γ^+^ CD8^+^ and CD4^+^ T cells are shown for sequential samples obtained after infection, PV1 and PV2 for **b**, **c** asymptomatic/mild (*n* = 6) and **d**, **e** moderate/severe (*n* = 11) groups. *P* values, calculated using Friedman’s test with Dunn’s multiple comparisons test, are shown above lines spanning the comparison groups when significant. Red lines bisecting groups represent median (solid) with IQR (dotted).

To gauge the capacity of SARS-CoV-2 S-specific T cells to proliferate and differentiate in vitro and to discriminate CD4^+^ and CD8^+^ T-cell responses, we assessed T-cell responses against SARS-CoV-2 S peptides by flow cytometry (Fig. 6a) following 7-day in vitro stimulation of PBMC from the same aliquot recovered to test circulating S-specific T cells. Restimulation of cultured cells from the asymptomatic/mild group (*n* = 6) at day 7 with S peptides activated up to 0.9% of CD8^+^ T cells (Fig. 6b) and up to 1.5% of CD4^+^ T cells (Fig. 6c) to produce IFN-γ. The mean percentage of SARS-CoV-2 S-reactive CD4^+^ or CD8^+^ T cells detected following in vitro expansion did not significantly increase in this group following either primary or secondary vaccination. Restimulation of cultured cells from the moderate/severe group (*n* = 11) at day 7 activated between 0.02 and 6.0% of CD8^+^ T cells (Fig. 6d) and between 0.04 and 10.0% of CD4^+^ T cells to produce IFN-γ (Fig. 6e). While the percentage of SARS-CoV-2 S-reactive CD4^+^ T cells detected following in vitro expansion did not significantly increase following either primary or secondary vaccination, the percentage of SARS-CoV-2 S-reactive CD8^+^ T cells significantly increased after first vaccination (Fig. 6d, *p* = 0.0417). There was an apparent, but non-statistically significant decline in the percentage of S-specific CD4^+^ T cells after secondary vaccination in this group (Fig. 6e). Infection with SARS-CoV-2 generates CD4^+^ and CD8^+^ T cell responses detectable by IFN-γ production that expand over 7 days in vitro with SARS-CoV-2 S peptide stimulation. Primary vaccination significantly boosted the frequency of S-specific CD8^+^ T cells detected after in vitro stimulation. Moderate or severe COVID-19 more effectively primes S-specific T cell, especially CD8^+^ T cell responses, to COVID-19 mRNA vaccines.

**Fig. 7:**
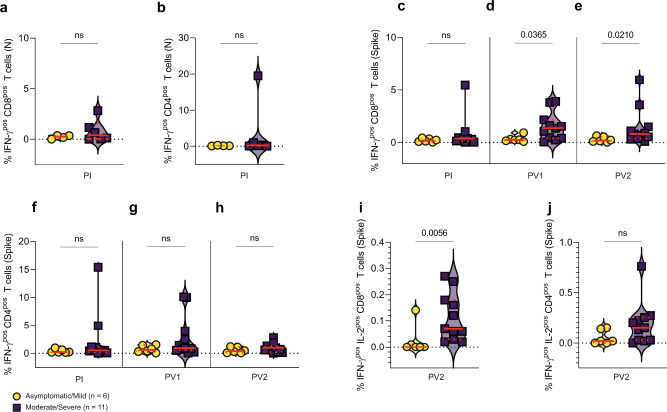
Comparison between asymptomatic/mild and moderate/severe infection groups of effector T-cell responses to SARS-CoV-2 N and S peptides following 7-day in vitro stimulation. **a** CD8^+^ T cell and **b** CD4^+^ T cell IFN-γ responses to N peptide restimulation from asymptomatic/mild (*n* = 4) and moderate/severe (*n* = 6) samples collected after infection were measured by flow cytometry after 7-day culture followed by 5 h restimulation. Similar comparisons of IFN-γ^+^ responses following S peptide restimulation were made for asymptomatic/mild (*n* = 6) and moderate/severe (*n* = 11) samples obtained **c** after infection, **d** PV1 and **e** PV2 for CD8^+^ T cells and **f** after infection, **g** PV1 and **h** PV2 for CD4^+^ T cells. Polyfunctional IFN-γ^+^/IL-2^+^ **i** CD8^+^ and **j** CD4^+^ T cell responses to S peptide restimulation are shown for asymptomatic/mild (*n* = 6) and moderate/severe (*n* = 11) groups. *P* values, calculated using the Mann–Whitney *U*-test, are shown above lines spanning the groups compared. Red lines bisecting groups represent median (solid) with IQR (dotted).

We next compared the magnitude of CD4^+^ and CD8^+^ T cell IFN-γ responses detected by flow cytometry after 7 days in vitro N or S peptide stimulation between the asymptomatic/mild and moderate/severe groups. Despite several participants in the moderate/severe infection group with between 0.5 and 2.9% of CD8^+^ T cells (Fig. 7a) and one participant with >20% of CD4^+^ T cells (Fig. 7b) producing IFN-γ in response to restimulation with SARS-CoV-2 N peptides pre-vaccination, there was no significant difference between the groups (Fig. 7a, b). Due to the nature of the vaccines (SARS CoV-2 S-encoding only), inducible SARS-CoV-2 N-specific responses were not measured again following primary or secondary vaccination. There was a similar distribution of responses against S peptides with comparable CD8^+^ T cell responses after infection (Fig. 7c), the difference between groups reaching significance after primary vaccination (Fig. 7d; *p* = 0.0365), and the significance remaining so after secondary vaccination (Fig. 7e; *p* = 0.0210). Two individuals in the moderate/severe group had >5% of their CD4^+^ T cells producing IFN-γ after restimulation with S peptides, however, the difference between CD4^+^ T cell responses of the asymptomatic/mild versus moderate/severe groups was not significant after first or second vaccination (Fig. 7f–h). More severe COVID-19 primed the immune system for stronger CD8^+^ T cell responses against SARS-CoV-2 S following vaccination.

While IFN-γ and TNF-α are important T-cell effector cytokines, IL-2 is critical for T-cell proliferation and long-term memory. Polyfunctional CD8^+^ IFN-γ^+^/IL-2^+^ (Fig. 7i, *p* = 0.0056), but not CD4^+^ (Fig. 7j, *p* = ns) T-cell responses against S peptides were significantly greater in the moderate/severe group compared with the asymptomatic/mild group. Only one participant in the asymptomatic/mild group (*n* = 6) had a polyfunctional CD8^+^ IFN-γ^+^/IL-2^+^ T cell response, whereas 5/11 in the moderate/severe cohort had >7.5% of their IFN-γ^+^ CD8^+^ T cells also producing IL-2 in response to S peptide restimulation (Fig. 7i). This included all 4 participants requiring hospitalization. Moderate/severe infection followed by vaccination selectively induced polyfunctional IL-2^+^/IFN-γ^+^ SARS-CoV-2 S-specific memory T cells that proliferated extensively in vitro following restimulation.

## Discussion

The severity of COVID-19 varies across a wide range, with age, co-morbidity and vaccination status the most significant influences^1–3^. Immunity against SARS-CoV-2 can provide protection against infection, or when breakthrough infections do occur, protection against illness^4,5^. As with other pathogens, SARS-CoV-2 mutates as it replicates and new variants are selected based on their cellular infectivity and host to host transmissibility. In the face of immunity from previous infection or vaccination, selection to evade pre-existing immunity also occurs. Thus, the efficacy of anti-SARS-CoV-2 immunity depends on the strength and breadth of specific Ab and T-cell responses. Mutations in the RBD region of SARS-CoV-2 S reduce the capacity of Ab induced by ancestral S antigens to neutralize contemporary SARS-CoV-2 variants, thereby affecting their breadth of activity. However, T-cell epitopes are largely conserved across ancestral and contemporary SARS-CoV-2 variants, thereby maintaining breadth of activity^17,18^. Persons who experience more severe illness tend to produce stronger immune responses, presumably through protracted exposure to the virus^34,35^. How this affects the strength and overall efficacy of vaccine-induced humoral and cellular immunity after first and second doses is an important question for gauging protection from future infection and illness.

Anti-S Ab levels rose significantly and substantially in the asymptomatic/mild and moderate/severe groups following one dose of vaccine, with those having experienced moderate or severe infection maintaining higher levels than those experiencing mild or no symptoms. The difference between groups was maintained following the second vaccine dose, despite it not producing a significant increase in Ab levels that lasted over ∼2.5 months in either group. These results illustrate the need for appropriate timing to maximize the benefits of immune boosting. In addition, the fact that Ab levels in the asymptomatic/mild group did not reach the same levels as in the moderate/severe group following two vaccinations suggests differential evolution towards response ceilings during infection. In this regard, one participant in the asymptomatic/mild group with no known immunocompromising condition had weak anti-S IgG Ab responses even after two vaccinations.

Differences observed in overall anti-FLS and anti-RBD IgG levels were reflected in the neutralization capacity of plasma Ab. More severe infection induced higher levels of neutralizing antibodies and vaccination boosted these levels in both groups. As higher levels of Ab against ancestral SARS-CoV-2 improve neutralization of contemporary variants, more severe infections should confer broader neutralization post-recovery and following downstream vaccination. Neutralization generally correlated very strongly with anti-FLS and anti-RBD levels measured by ELISA, but in certain cases, neutralization was less than would be expected based on ELISA values. Therefore, there is individual qualitative variability in IgG Ab raised against FLS and RBD in terms of fine specificity or avidity required to neutralize infection. This could potentially leave some individuals with relatively high anti-RBD and anti-FLS IgG Ab levels at risk for infection or illness.

Antibodies against SARS-CoV-2 S protein can also mediate antiviral effects through Fc receptor-dependent mechanisms. Persons with moderate/severe COVID-19 had high levels of ADCC-inducing Ab when tested shortly after infection and vaccination boosted levels of these Ab. As with neutralizing Ab, levels of ADCC-inducing Ab against SARS-CoV-2 generally decline with time since infection. Our results from this study reflect cross-sectional comparison of ADCC levels, while longitudinal analysis would more directly assess waning of ADCC capacity. Mechanisms such as ADCC may be important in limiting illness by eliminating infected host cells when infections do occur, especially with conservation of ADCC targets across variants^12^. Other Fc receptor-dependent mechanisms such as antibody-dependent enhancement (ADE) can have pro-viral and/or pro-inflammatory effects that worsen illness^36–38^. The overall role of non-neutralizing Ab against SARS-CoV-2 S has not been established, but the potential for ADE increases with viral evolution away from neutralization by Ab induced through previous infection or by vaccination with ancestral strains.

In a previous study comparing subjects with mild (*n* = 4) versus severe (*n* = 4) versus critical (*n* = 1) illness, analysis of B and T cell receptor sequences in PBMC demonstrated more pronounced expansion of SARS-CoV-2-specific B and T cell clones with more severe illness^34^. Functional analysis of T cell responses against a genome-wide set of predicted epitopes also indicated a higher frequency of IFN-γ-secreting T cells specific for SARS-CoV-2 with greater differentiation towards a terminal effector phenotype in subjects with severe illness^34^. We compared immunity against SARS-CoV-2 induced by asymptomatic or mild infection to that induced by moderate or severe infection after recovery from infection and in the same subjects, after primary and secondary doses of approved COVID-19 vaccines. Based on the nature of the vaccines, we focused primarily on antibody levels and functions against SARS-CoV-2 spike protein together with measuring the frequency and nature of SARS-CoV-2 spike-specific T cells. This enabled assessment of the impact of severity of infection initially on infection-acquired immunity and downstream on vaccine-induced immunity against SARS-CoV-2. In agreement with the previous study, following infection we detected higher Ab levels against SARS-CoV-2 S and N antigens and more robust T cell responses in the group with more severe infections and extended our study through primary and secondary vaccination to demonstrate more robust hybrid immunity following vaccination^34^.

We compared cellular immune responses against SARS-CoV-2 for a subset of subjects in each group. Circulating cellular immune responses against S followed a similar pattern to that seen for humoral responses in that they were stronger post infection in the group with moderate or severe infection and remained stronger through initial and secondary boosting by vaccination. Like humoral immune responses, primary, but not secondary vaccination significantly boosted circulating cellular immune responses in both groups. As with the humoral immune response, previous infection primes the immune system for cellular responses, but may also set limits on the strength of immune responses induced following SARS-CoV-2 vaccination as the responses of the asymptomatic/mild group remained lower than those of the moderate/severe group through two rounds of vaccination. Inducible cellular immune responses against SARS-CoV-2 S protein were not significantly affected by vaccination, but following primary vaccination, the inducible CD8^+^ T cell response was stronger in the group experiencing moderate/severe infection.

Some ambiguity between results reported from different studies measuring T cell responses against SARS-CoV-2 is likely based in part on different methodology. One study reported lesser detection of cross-reactive responses against common coronavirus with IFN-γ ELISpot assays compared to other detection methods^20^. Although the relationship between intracellular cytokine production and expression of immune activation markers following stimulation is proportional, intracellular cytokine production is less frequent than immune activation markers, which may accentuate differences between studies^39^. Bystander T cell receptor-independent activation selectively affects memory helper T cells, potentially skewing responses towards CD4^+^ T cell domination^40^. A previous study using an equivalent peptide set to stimulate circulating T cells for 15 h and detecting responses by activation marker expression and cytokine production, reported a dominant CD4^+^ T cell response against SARS-CoV-2 following recovery from infection or vaccination, that was boosted by vaccination. We measured circulating responses by IFN-γ ELISpot without distinguishing CD4^+^ from CD8^+^ T cells. After 7 days of in vitro stimulation, we observed individual variability in CD4^+^ and CD8^+^ T cell dominance within the responding T cell populations that expanded in culture. This may reflect individual differences in the subjects or overrepresentation of bystander activated CD4^+^ T cells following stimulation of freshly isolated PBMC. Bystander activated T cells are more susceptible to apoptosis than those clonally activated through T cell receptors and, therefore, less likely to selectively proliferate over longer term culture^40^.

Inducible cellular immune responses measured in vitro against SARS-CoV-2 reflect reactivation and expansion of memory T cells. Mild/asymptomatic infection appears to establish stable, responsive CD4^+^ and CD8^+^ memory T cells that potentially provide protection against severe illness. Expansion of S-specific T cells over 7 days in vitro may tend to equalize responses between groups, although several outliers in the moderate/severe group had especially high frequencies of S-specific CD4^+^ and CD8^+^ T cells. The frequency of inducible CD8^+^ T cells against SARS-CoV-2 S in the moderate/severe group was boosted by primary vaccination to higher levels than in the asymptomatic/mild group, especially when considering polyfunctional IL-2^+^/IFN-γ^+^ CD8^+^ T cells. It would be informative to compare memory subset status of circulating SARS-CoV-2-specific T cells in both groups, but in most cases, the frequency is too low to reliably assess this without large numbers of PBMC. The general stability of T-cell responses post infection and vaccination independent of the severity of infection is consistent with retaining longer term protection against severe illness than against infection itself through previous infection and/or vaccination. However, the significant boosting of inducible SARS-CoV-2 S-specific CD8^+^ T cell responses selectively observed following vaccination of subjects experiencing moderate or severe COVID-19 suggests there may be a long-term immunological advantage associated with recovery from more severe COVID-19.

The apparent plateauing of immune responses at different heights in both groups with only marginal responses to secondary vaccination suggests response limits are related to either inherent host factors or host factors conditioned through the course of initial infection. While immune system priming through previous infection has a positive effect on hybrid immunity against SARS-CoV-2, pre-existing immunity against the backbone of recombinant viruses can reduce responses against the vaccine insert^41,42^. Whether this is an issue for RNA vaccines and if so, how to optimize vaccine regimens accordingly requires investigation. Third, fourth and fifth boosts with RNA vaccines are new ground and the role pre-existing immunity plays in boosting itself versus accelerating vaccine clearance is under-investigated. A vaccine formulation that does not engage CD8^+^ T cells might more effectively boost humoral responses in the face of pre-existing humoral immunity. It is critical to better understand the immunology of protection from infection and protection from illness as we move towards the development of more effective and durable vaccine regimens against SARS-CoV-2. If neutralizing mucosal Ab protect against infection, while ADCC and T-cell responses mediate clearance and protection from severe illness, then the greater breadth of the latter responses against multiple SARS-CoV-2 variants should elevate their priority for targeting by vaccines. Determining the appropriate vaccine for an individual ideally requires analysis of their pre-existing humoral and cellular immunity. Given the short-term protection against contemporary infection offered by mRNA-based boosting with ancestral SARS-CoV-2 strains, it may be possible to define a limit below which humoral immunity is ineffective at providing broad protection. Developing feasible strategies to maintain Ab levels above this limit, in combination with better understanding the cellular immune mechanisms contributing protection from illness is a path towards providing broad and durable protection against contemporary and emerging SARS-CoV-2 variants.

In summary, a group of individuals who experienced asymptomatic or mild infection with SARS-CoV-2 sustained weaker immune responses against SARS-CoV-2 through two rounds of vaccination compared to a group of individuals who experienced moderate or severe infection. Although both groups responded robustly to initial vaccination, responses to secondary boosting were insignificant approximately 2.5 months post-vaccination. Several previous studies reported similar findings with Ab measurements earlier after vaccination, including no increase in neutralization titre^27,29,32^. The finding that both groups’ responses plateaued with the second vaccination illustrates the importance of timing to optimize vaccine responses and also illustrates the risk of categorizing all potential vaccinees as a single group, irrespective of their infection history. Another study did report increases in Ab levels following secondary vaccination in persons recovering from asymptomatic or paucisymptomatic infection, but not in those recovered from more severe infection^30^. Ironically, with lower levels of starting immunity waning over time and lesser expansion of CD8^+^ T memory cells, people who dealt with their initial exposure to SARS-CoV-2 most readily might be at greater risk of illness upon subsequent exposure, especially to a variant, while those who suffered severe infection are protected. With broad uptake of COVID-19 vaccines throughout certain countries, widespread Omicron infections and ongoing introduction of new vaccines, immune status should be considered in ascribing vaccine regimens, rather than imposing a “one size fits all approach” that might fail some and be wasted on others.

## Methods

### Study subjects

This study was carried out in accordance with recommendations of the Canadian Tri-Council Policy Statement: Ethical Conduct for Research Involving Humans. Protocols to obtain peripheral blood from persons with confirmed or suspected SARS-CoV-2 infection were approved by the Health Research Ethics Authority of Newfoundland and Labrador (HREA). Participants were recruited through news and social media, poster placement, and word of mouth. A questionnaire addressing previous testing history and reasons for suspecting infection with SARS-CoV-2 was administered at study intake and peripheral blood was collected from study subjects by forearm venipuncture at ∼three-month intervals after obtaining written informed consent in accordance with the Declaration of Helsinki. Persons with previous RT-PCR-confirmed SARS-CoV-2 infection or who suspected previous infection, which was subsequently confirmed serologically, were recruited to study immune responses against SARS-CoV-2^19^. Thirty-five individuals with confirmed infection who continued in the study and received 2 doses of a COVID-19 vaccine were categorized blindly as experiencing asymptomatic (score = 0) mild (score = 1), moderate (score = 2) or severe (score = 3) COVID-19 based on the duration and intensity of their self-reported symptoms. This cohort was matched with 35 participants with no SARS-CoV-2 infection history who received 2 doses of a COVID-19 vaccine to compare humoral responses. Participants self-declared any medical treatments they were receiving as well as information on comorbidities. Persons with any known underlying immune compromising condition or on immunosuppressive treatment were excluded from this study.

### Sample processing

Whole blood was collected by venipuncture into acid citrate dextrose solution A containing vacutainers. Plasma was collected following 10 min centrifugation at 500 × *g* and stored at −80 °C and PBMC were isolated using the Canadian Autoimmunity Standardization Core consensus standard operating procedure (version: March 21, 2019). Freshly isolated PBMC were resuspended in freezing medium consisting of fetal bovine serum (FBS; HyClone™, GE Healthcare Life Sciences, Logan UT, USA) supplemented to 10% dimethyl sulfoxide (DMSO; Sigma-Aldrich, St. Louis MO, USA) and cooled at 1 °C per minute overnight to −80 °C then maintained in liquid nitrogen until use.

### Serological testing

Plasma diluted 1:500 in PBS containing 0.05% TWEEN® 20 (Sigma-Aldrich, St. Louis, MO, USA) and 0.1% bovine serum albumin (BSA, Sigma-Aldrich) was tested against recombinant proteins coated at 50 ng/well in Dulbecco’s PBS (DPBS, Sigma-Aldrich) overnight (50 μL/well) onto 96-well Immunlon-2 plates (VWR Scientific, Mississauga, ON, Canada). Recombinant protein antigens included the RBD of SARS-CoV-2 S protein (SinoBiological, Wayne, PA, USA), full-length SARS-CoV-2 S glycoprotein trimer [SMT1-1, National Research Council (NRC), Canada] and SARS-CoV-2 N protein (SinoBiological). Diluted plasma (100 μL/well) was applied to antigen-coated plates after 1 h block (200 μL/well) with 1% BSA in PBS followed by six washes using wash buffer (PBS containing 0.05% TWEEN® 20) and binding of specific IgG detected with a 1:50 000 dilution (100 μL/well) of HRP-conjugated polyclonal goat anti-human IgG (Jackson ImmunoResearch Labs). Plates were washed six times with wash buffer followed by the addition of TMB substrate (Sigma-Aldrich; 100 μL/well). Reactions were stopped after 20 minutes of color development using an equal volume of 1 M H_2_SO_4_ (Sigma-Aldrich) and optical density (OD) was read on a BioTek synergy HT plate reader at 450 nm^19^. A human IgG_1_ neutralizing Ab against RBD, mAb SAD-S35 (AcroBiosystems, Newark, DE, USA), was used at 16 ng/mL for SMT1-1-coated conditions and 8 ng/mL (100 μL/well) for RBD-coated conditions on each plate to reach OD ~1 and OD readings from test plasma samples were normalized to these values.

### Cell culture

All cell lines and PBMC were cultured with 5% CO_2_ at 37 °C. Vero (K. Hirasawa, Memorial University), MRC-5 (ATCC® CCL-171™), and Lenti-X™ 293T cells (TakaRa, Mountain View CA, USA) were propagated in complete DMEM (Sigma-Aldrich) containing 10% FBS (HyClone™) and 200 IU/mL penicillin/streptomycin (Invitrogen, Carlsbad CA, USA). PBMC were recovered overnight in lymphocyte medium consisting of RPMI-1640 with 10% FBS (HyClone™), 200 IU/mL penicillin/streptomycin, 0.01 M HEPES, 1% L-glutamine (all from Invitrogen) and 2 × 10^−5^ M 2-mercaptoethanol (Sigma-Aldrich) and used in functional experiments when viability >75%, determined by trypan blue staining.

### SARS-CoV-2 neutralization assay

SARS-CoV-2 antibody neutralization was quantified in a virus-based assay using replication-competent recombinant vesicular stomatitis virus (rVSV) expressing wild-type Wuhan-Hu-1 spike with eGFP (rVSV-S; BEI Resources)^43,44^. Vero cells (10^4^/well) were seeded overnight in 96-well flat-bottom plates (Corning™) to obtain ~ 80–90% confluency. Plating medium was removed, and heat-inactivated (56 °C for 1 h) plasma, diluted by 11 two-fold dilutions in DMEM containing 2% FBS (HyClone™) and 200 IU/mL penicillin/streptomycin (Invitrogen), was applied in duplicate and incubated with 5% CO_2_ at 37 °C for 30 min. An equal volume of rVSV-S was added at MOI 0.1 and cultured with 5% CO_2_ at 37 °C for 24 h at which time the medium was decanted and eGFP positive cells enumerated using the CTL ImmunoSpot® S6 Universal Analyzer (C.T.L Analyzers, Shaker Heights, OH, USA). Percent neutralization was calculated as (eGFP positive cells with no plasma) − (eGFP positive cells at each plasma dilution)/(eGFP positive cells with no plasma) × 100%. The half-maximal inhibitory concentration (IC_50_) was calculated from these values by nonlinear regression analysis with GraphPad Prism Version 9.3.0 and inverse log_10_ values plotted.

### Wuhan-Hu-1 Spike expression in MRC-5 cells

The recombinant Lenti-X™ pLVX-IRES lentiviral vector expression system (TakaRa) was used to introduce the Wuhan-Hu-1 spike sequence into the human lung fibroblast MRC-5 cell line. Briefly, the canonical Wuhan-Hu-1 spike sequence was obtained from BEI Resources in a pcDNA™ 3.1(-) mammalian expression vector (NR-52420; NIAID, NIH)^45^ and inserted into pLVX-IRES after XbaI and BamHI restriction digestion [New England Biolabs® (NEB®), Ipswich, MA, USA] and agarose gel extraction/purification (QIAquick Gel Extraction; Qiagen, Toronto, ON, Canada). The pLVX-IRES/ Wuhan-Hu-1 spike expression vector was transformed into high efficiency 10-beta competent E. coli (NEB®) from which midi-scale plasmid DNA was prepared (NucleoBond Xtra Midi, TakaRa), concentration determined using NanoDrop™ (Thermo Fisher Scientific) and sequenced (positive and negative strands; TCAG Facilities, Hospital for Sick Children, Toronto ON, Canada) to ensure authenticity. SnapGene® 5.3.2 (San Diego CA, USA) was used for designing and visualizing cloning procedures and designing and aligning sequencing primers. Lenti-X™ 293T cells were transfected with the pLVX-IRES/Wuhan-Hu-1 spike expression vector using the Lenti-X™ Single Shot (TakaRa) packaging and transfection system. Lentiviral supernatants were collected 48 h after transfection, filtered through a 0.45 μm polyethersulfone filter to remove cellular debris, aliquoted, and frozen at −80 °C. A p24 ELISA [Leidos Biomedical Research, Inc., for the National Cancer Institute (NCI), Frederick, MD, USA], read at 450 nm on a Synergy HT BioTek microplate reader, was used to obtain viral titres and 7 × 10^5^ MRC-5 cells plated overnight in 12-well plates were transduced with lentiviral supernatant and 4 μg/mL polybrene (Sigma-Aldrich) by 90 min 1200 × *g* spinoculation at 32 °C. Transduction medium was replaced 24 h later with complete DMEM, cells were propagated and selected in complete DMEM containing 0.625 μg/mL puromycin (Sigma-Aldrich). To confirm extracellular spike expression, non-transduced or S-transduced MRC-5 cells were lifted with Accutase® (STEMCELL™ Technologies, Vancouver BC, Canada) as per manufacturer’s instructions, stained for 20 min at room temperature using a 1:100 dilution of heat-inactivated SARS-CoV-2 seropositive plasma followed by 1:200 dilution of goat anti-human IgG Fc-PE secondary antibody (eBioscience, San Diego, CA, USA). Data were acquired using the CytoFLEX flow cytometer and analyzed and illustrated using Kaluza Version 2.1 (both Beckman Coulter, Brea CA, USA).

### Antibody-dependent cell-mediated cytotoxicity assay

PBMC were freshly processed (as above) on the day of experiment, resuspended in lymphocyte medium, and kept at 37 °C and 5% CO_2_ until use. Non-transduced or Wuhan-Hu-1 S-expressing MRC-5 target cells (10^4^/well) were plated and labeled with 1 μCi Na_2_^51^CrO_4_ (PerkinElmer, Akron, OH, USA) per well overnight in 96-well round bottom plates. Target cells were washed four times in PBS containing 1% FBS (HyClone™) then PBMC (E:T 25:1) and heat-inactivated plasma was added to wells with a final volume (*V*_f_) of 300 μL and final dilution of 1:1000. ADCC activity from each plasma donor was measured using PBMC from healthy donors with previously characterized ADCC activity and cytotoxicity was measured by ^51^Cr release over 5 h. ^51^Cr release was measured in 125 μL supernatant on a Wallac 1480 Wizard gamma counter and percent specific lysis calculated by (experimental ^51^Cr release − spontaneous ^51^Cr release)/(maximum ^51^Cr release − spontaneous ^51^Cr release) × 100.

### Peptide pool preparation and IFN-γ ELISpot assay

The peptide pool for Wuhan-Hu-1 S (PepTivator® SARS-CoV-2 Prot_S Complete; Miltenyi Biotec, San Diego CA, USA) consisting of 15-mers with 11 amino acid (aa) overlap covering functional domains (aa 5–1273) of the S sequence was reconstituted at 50 μg/mL in endotoxin-free ultra pure water (EF H_2_0; Sigma-Aldrich) and individual peptides comprising the N peptide array (NR-52404; BEI Resources) consisting of 17- or 13-mers with 10 aa overlaps were reconstituted at 10 mg/mL in DMSO (Sigma-Aldrich), then pooled. Acute T-cell responses were measured using IFN-γ enzymatic ELISpot (ImmunoSpot®, Cleveland OH, USA) designed for 96-well polyvinylidene fluoride membrane-bottomed plates as outlined by the manufacturer. Vehicle controls of EF H_2_0 and DMSO were included to obtain background IFN-γ signals and anti-CD3 (OKT3, ATCC® CRL-8001) used as positive control. Spike and N peptide pools were used at a final concentration of 1 μg of each peptide/mL to stimulate 2 × 10^5^ PBMC (*V*_f_ = 200 μL) for 24 h. Cells and peptides or cells and anti-CD3 were diluted in CTL-Test™ Medium (ImmunoSpot®) and each condition was performed in duplicate. Background (vehicle) spot counts were subtracted from S or N peptide pool stimulated spot counts and IFN-γ^+^ spot forming cells per million PBMC (IFN-γ^+^ SFC/10^6^ PBMC) captured on a CTL ImmunoSpot® S6 Universal Analyzer and illustrated using GraphPad Prism Version 9.3.0.

### Detection of S-specific cytokine production by intracellular flow cytometry

SARS-CoV-2 T-cell responders were chosen based on IFN-γ responses detected by 24 h ELISpot data and PBMC from the same recovered aliquot were stimulated for 7 days when sufficient remaining cells were available. A total of 2–5 × 10^6^ PBMC were stimulated with S (Miltenyi Biotec) or N (BEI Resources) peptide pools at 0.5 μg of each peptide/mL for 1 h in a minimal volume after which time 1 mL of lymphocyte medium containing 25 ng/mL of IL-7 (NCI) was added and cell culture continued. Lymphocyte medium was added as necessary from day 3. On day 6, cultures were counted and resuspended at 2 × 10^6^ cells/mL in lymphocyte medium. Effector T cells (0.5 × 10^6^ cells) were restimulated for 5 h on day 7 with 0.5 μg/mL of S (Miltenyi Biotec) or N (BEI Resources) peptide pools in the presence of 10 μg/mL Brefeldin A (Sigma-Aldrich) in a *V*_f_ of 500 μL. Effector T cells were stained with directly conjugated mAb (conjugate, clone in parentheses) against human CD3 (VioGreen, REA613) and CD4 (APC-Vio770, REA623) from Miltenyi Biotec, CD8 (AlexaFluor® 700, HIT8a) from BioLegend, and intracellular IFN-γ (APC, 4 S.B3), IL-2 (PE, MQ1-17H12) from Invitrogen and TNF-α (Brilliant Violet 421™, MAb11) from BioLegend using the Inside Stain Kit (Miltenyi Biotec) as per manufacturer’s instructions. Data were acquired on a CytoFLEX flow cytometer and analyzed and illustrated using Kaluza Version 2.1 (both Beckman Coulter) and GraphPad Prism Version 9.3.0. Intracellular IL-2 and IFN-γ in CD4^+^ or CD8^+^ T cells responding to peptide restimulation on day 7 of culture was detected by flow cytometry with the signal from non-restimulated cells subtracted as background.

### Statistical analysis

Statistical analyses were performed using GraphPad Prism Version 9.3.0 with two-sided *p*-values < 0.05 considered significant. The normality of data distribution was assessed using Shapiro–Wilk test. Significance of correlations were assessed using Spearman’s rank correlation coefficient. Differences in means with standard deviation (SD) or medians with interquartile range (IQR, calculated as IQR = Q_3_ − Q_1_) between groups were compared using one-way ANOVA, Student’s *t*-test or Mann–Whitney *U*-test as appropriate based on normality of data distribution.

### Reporting summary

Further information on research design is available in the Nature Research Reporting Summary linked to this article.

## Supplementary information

REPORTING SUMMARY

## Data Availability

Data supporting the findings of this study and preserving anonymity of study participants will be made available from the corresponding authors through electronic correspondence for legitimate scientific purposes.
