# Supplementary material for: Moderate to severe SARS-CoV-2 infection primes vaccine-induced immunity more effectively than asymptomatic or mild infection
Source: NPJ Vaccines. 2022 Oct 21;7:122. doi: 10.1038/s41541-022-00546-1 (PMC9587265; doi:10.1038/s41541-022-00546-1)
Supplement: Supplementary file 1 — REPORTING SUMMARY [file 41541_2022_546_MOESM1_ESM.pdf]

nature portfolio

Corresponding author(s): Dr. Michael Grant, Dr. Kayla Holder

Last updated by author(s): Sep 2, 2022

Reporting Summary

Nature Portfolio wishes to improve the reproducibility of the work that we publish. This form provides structure for consistency and transparency in reporting. For further information on Nature Portfolio policies, see our Editorial Policies and the Editorial Policy Checklist.

Statistics

For all statistical analyses, confirm that the following items are present in the figure legend, table legend, main text, or Methods section.

n/a	Confirmed	
☐	☒	The exact sample size (*n*) for each experimental group/condition, given as a discrete number and unit of measurement
☐	☒	A statement on whether measurements were taken from distinct samples or whether the same sample was measured repeatedly
☐	☒	The statistical test(s) used AND whether they are one- or two-sided<br>*Only common tests should be described solely by name; describe more complex techniques in the Methods section.*
☐	☒	A description of all covariates tested
☐	☒	A description of any assumptions or corrections, such as tests of normality and adjustment for multiple comparisons
☐	☒	A full description of the statistical parameters including central tendency (e.g. means) or other basic estimates (e.g. regression coefficient) AND variation (e.g. standard deviation) or associated estimates of uncertainty (e.g. confidence intervals)
☐	☒	For null hypothesis testing, the test statistic (e.g. *F*, *t*, *r*) with confidence intervals, effect sizes, degrees of freedom and *P* value noted<br>*Give P values as exact values whenever suitable.*
☒	☐	For Bayesian analysis, information on the choice of priors and Markov chain Monte Carlo settings
☒	☐	For hierarchical and complex designs, identification of the appropriate level for tests and full reporting of outcomes
☐	☒	Estimates of effect sizes (e.g. Cohen's *d*, Pearson's *r*), indicating how they were calculated

Our web collection on statistics for biologists contains articles on many of the points above.

Software and code

Policy information about availability of computer code

Data collection	CTL ImmunoSpot® S6 Universal Analyzer was used to enumerate ELISpot.
Data analysis	Serology data was normalized using Microsoft Excel v2206.<br>Nonlinear regression analysis was performed using GraphPad Prism Version 9.3.0 to plot neutralization data. All graphs were plotted and statistical analysis performed with GraphPad Prism Version 9.3.0.<br>SnapGene® 5.3.2 was used for cloning strategy and sequencing alignment.<br>Kaluza Version 2.1 was used to analyze .fcs files.

For manuscripts utilizing custom algorithms or software that are central to the research but not yet described in published literature, software must be made available to editors and reviewers. We strongly encourage code deposition in a community repository (e.g. GitHub). See the Nature Portfolio guidelines for submitting code & software for further information.

Data

Policy information about availability of data

All manuscripts must include a data availability statement. This statement should provide the following information, where applicable:

- Accession codes, unique identifiers, or web links for publicly available datasets
- A description of any restrictions on data availability
- For clinical datasets or third party data, please ensure that the statement adheres to our policy

Data that support the findings of this study will be made available from the corresponding author upon reasonable request.

Human research participants

Policy information about studies involving human research participants and Sex and Gender in Research.

Reporting on sex and gender	Human research participants were categorized by sex at birth for all reporting.
Population characteristics	Ethnic characteristics were not collected from study participants.
Recruitment	Participants were recruited through news and social media, poster placement and word of mouth. A questionnaire addressing previous testing history and reasons for suspecting infection with SARS-CoV-2 was administered at study intake and peripheral blood was collected from study subjects by forearm venipuncture.
Ethics oversight	Health Research Ethics Authority of Newfoundland and Labrador (HREA)

Note that full information on the approval of the study protocol must also be provided in the manuscript.

Field-specific reporting

Please select the one below that is the best fit for your research. If you are not sure, read the appropriate sections before making your selection.

☒ Life sciences ☐ Behavioural & social sciences ☐ Ecological, evolutionary & environmental sciences

For a reference copy of the document with all sections, see nature.com/documents/nr-reporting-summary-flat.pdf

Life sciences study design

All studies must disclose on these points even when the disclosure is negative.

Sample size	No statistical methods were used to select sample size. There were 39 participants who had infection followed by two doses of vaccination at the time we determined cohorts.
Data exclusions	Data were excluded when participants were determined to be immune compromised or on immunosuppressive treatment.
Replication	MRC-5 cell phenotying after spike transduction was performed on different passages and different days.<br>Participant samples are longitudinal and although some samples will be used for future comparisons, others no longer exist and replication cannot be determined. Participants were grouped based on symptoms to reduce incidence of spurious outcomes.
Randomization	We used criteria for mild, moderate and severe infection based on self-perceived symptom intensity over longer or shorter period of times, which we believe would reflect different levels of immune system stimulation. Clinical criteria from hospitalization and treatment were not available for this study, but we considered any case where hospitalization was required as severe. Duration of infection and symptoms experienced were collected from symptomatic persons. We blinded the group and assigned them a disease severity score (0 asymptomatic, 1 mild, 2 moderate, 3 severe) based on listed symptoms and duration of these symptoms to limit degree of bias. Most common symptoms included fever, cough, tiredness, loss of taste or smell, sore throat, headache, aches and pains and are reflected in the majority of participants scored for severity.
Blinding	The group was blinded to assign disease severity, however, all samples were labeled with a unique identifier. Samples used were tested in numerical order and grouped based on symptoms after analysis.

Reporting for specific materials, systems and methods

We require information from authors about some types of materials, experimental systems and methods used in many studies. Here, indicate whether each material, system or method listed is relevant to your study. If you are not sure if a list item applies to your research, read the appropriate section before selecting a response.

Materials & experimental systems

n/a	Involved in the study	
☐	☒	Antibodies
☐	☒	Eukaryotic cell lines
☒	☐	Palaeontology and archaeology
☒	☐	Animals and other organisms
☒	☐	Clinical data
☒	☐	Dual use research of concern

Methods

n/a	Involved in the study	
☒	☐	ChIP-seq
☐	☒	Flow cytometry
☒	☐	MRI-based neuroimaging

Antibodies

Antibodies used

HRP-conjugated polyclonal goat anti-human IgG; Jackson ImmunoResearch Labs; Catno. 111-035-144; Lot no. 148901
anti-human SAD-S35 IgG1; AcroBiosystems; Clone/Cat no. SAD-S35; Lot no. S35-20AVF1-U8
anti-human IgG Fc PE; eBioscience; Cat no. 12-4998-82; Lot no. 2329679
anti-human CD3; ATCC; Clone OKT3; Cat no. CRL-8001™
anti-human CD3 viogreen; Miltenyi Biotech; Clone REA613; Cat no. 130-113-142; Lot no. 5210707645
anti-human CD4 APC-Vio770; Miltenyi Biotech; Clone REA623; Cat no. 130-113-223; Lot no. 5211205270
anti-human CD8a AlexaFluor® 700; BioLegend; Clone HIT8a; Cat no. 300920; Lot no. B347057
anti-human IFN-γ APC; Invitrogen; Clone 4S.B3; Cat no. 14-7319-81; Lot no. 2344999
anti-human IL-2 PE; Invitrogen; Clone MQ1-17H12; Cat no. 14-7029-81; Lot no. 2489240
anti-human TNF-α Brilliant Violet 421™; BioLegend; Clone MAb11; Cat no. 502932; Lot no. B238842

Validation

Commercially-available antibodies were validated by manufacturer. Anti-human CD3 (OKT3) was validated by flow cytometry and CD3 stimulation.

Eukaryotic cell lines

Policy information about cell lines and Sex and Gender in Research

Cell line source(s)

293T - female human embryonic kidney
MRC-5 - male fetal lung fibroblast
Vero - adult female African green monkey kidney epithelial

Authentication

293T and MRC-5 cell lines were authenticated from their commercial source. Vero cells were not authenticated.

Mycoplasma contamination

Cell lines were not tested for Mycoplasma contamination.

Commonly misidentified lines
(See ICLAC register)

None used.

Flow Cytometry

Plots

Confirm that:

☒ The axis labels state the marker and fluorochrome used (e.g. CD4-FITC).

☒ The axis scales are clearly visible. Include numbers along axes only for bottom left plot of group (a 'group' is an analysis of identical markers).

☒ All plots are contour plots with outliers or pseudocolor plots.

☒ A numerical value for number of cells or percentage (with statistics) is provided.

Methodology

Sample preparation

Whole blood was collected from study participants and peripheral blood mononuclear cells isolated by ficoll-hypaque density gradient centrifugation. Cells not used immediately after isolation in assays were cryopreserved in 90% fetal calf serum, 10% dimethylsulphoxide in liquid N2 as described in materials and methods. Cryopreserved cells were thawed and treated as described in materials and methods before analysis.

Instrument

Beckman Coulter CytoFlex Violet-Blue-Red (V2-B5-R2) Series Flow Cytometer (model B50946)

Software

CytExpert v2.5 was used to collect .fcs data and Kaluza Version 2.1 was used to analyze .fcs files.

Cell population abundance

Cell populations were not sorted prior to analysis.

Gating strategy

Lymphocytes were identified by scatter characteristics and doublet exclusion, T cells identified as CD3+ lymphocytes and subsets distinguished by either CD4 or CD8 expression. Bivariate analysis identified subsets of CD4+ T cells and CD8+ T cells

producing TNF-α, IFN-γ and IL-2 after restimulation at day 7 and background signals from non-restimulated cells were subtracted.

☒ Tick this box to confirm that a figure exemplifying the gating strategy is provided in the Supplementary Information.
